# Ancient DNA Resolves the History of *Tetragnatha* (Araneae, Tetragnathidae) Spiders on Rapa Nui

**DOI:** 10.3390/genes8120403

**Published:** 2017-12-21

**Authors:** Darko D. Cotoras, Gemma G. R. Murray, Joshua Kapp, Rosemary G. Gillespie, Charles Griswold, W. Brian Simison, Richard E. Green, Beth Shapiro

**Affiliations:** 1 Department of Ecology and Evolutionary Biology, University of California, Santa Cruz, 1156 High Street, Santa Cruz, CA 95064, USA; ggrmurray@gmail.com (G.G.R.M.); jkapp@ucsc.edu (J.K.); bashapir@ucsc.edu (B.S.); 2 Entomology Department, California Academy of Sciences, 55 Music Concourse Dr., Golden Gate Park, San Francisco, CA 94118, USA; CGriswold@calacademy.org; 3 Center for Comparative Genomics, California Academy of Sciences, 55 Music Concourse Dr., Golden Gate Park, San Francisco, CA 94118, USA; BSimison@calacademy.org; 4 Department of Environmental Science, University of California, 137 Mulford Hall, Berkeley, CA 94720-3114, USA; gillespie@berkeley.edu; 5 Department of Biomolecular Engineering, University of California, Santa Cruz, 1156 High Street, Santa Cruz, CA 95064, USA; ed@soe.ucsc.edu

**Keywords:** arachnid, museum, ancient DNA, Rapa Nui, *Tetragnatha*

## Abstract

Rapa Nui is one of the most remote islands in the world. As a young island, its biota is a consequence of both natural dispersals over the last ~1 million years and recent human introductions. It therefore provides an opportunity to study a unique community assemblage. Here, we extract DNA from museum-preserved and newly field-collected spiders from the genus *Tetragnatha* to explore their history on Rapa Nui. Using an optimized protocol to recover ancient DNA from museum-preserved spiders, we sequence and assemble partial mitochondrial genomes from nine *Tetragnatha* species, two of which were found on Rapa Nui, and estimate the evolutionary relationships between these and other *Tetragnatha* species. Our phylogeny shows that the two Rapa Nui species are not closely related. One, the possibly extinct, *T. paschae*, is nested within a circumtropical species complex (*T. nitens*), and the other (*Tetragnatha* sp. Rapa Nui) appears to be a recent human introduction. Our results highlight the power of ancient DNA approaches in identifying cryptic and rare species, which can contribute to our understanding of the global distribution of biodiversity in all taxonomic lineages.

## 1. Introduction

Rapa Nui, or Easter Island, is one of the most isolated islands on Earth. Located in the easternmost corner of the Polynesian triangle, this subtropical island is 3500 km from the Chilean coast and 4500 km from Tahiti. The island is both small (163,61 km^2^) and young; it is comprised of three volcanoes—Poike, Maunga Terevaka, and Rano Kau—that range in age from 0.94 to 0.11 million years old [1,2]. Rapa Nui’s biota reflects a unique combination of species that are likely the result of long-distance dispersal from both Asia and America over the last million years, including many recent human introductions [3]. The biodiversity of the island has also been profoundly affected by ecological shifts that are driven by the complete deforestation of the island between Polynesian colonization (around 1200 AD [4]) and European contact (1722) [5,6], and by the use of the island for intensive sheep grazing over the first half of the 20th century [7]. This ecological collapse has left an impoverished arthropod fauna, where only one spider has been described as endemic, *Tetragnatha paschae* [8,9]. 

The genus *Tetragnatha* has a global distribution [10], and has colonized many remote archipelagoes [11]. These spiders are often among the first colonizers after habitat perturbations [12], and they have been extensively studied as a model system for adaptive radiations on islands [11]. For example, a clade of the Hawaiian species has moved into rainforests, and have become active hunters, abandoning the riparian habitat and “sit-and-wait” on an orb-web hunting strategy that is shared among other *Tetragnatha* [13]. Three species of *Tetragnatha* spiders have been reported on Rapa Nui: *Tetragnatha nitens*, *Tetragnatha paschae*, and one whose identity has yet to be established. *T. nitens* has a circumtropical distribution, and has been recorded on Rapa Nui in three separate surveys of the island [9]: a Swedish expedition in 1916–1917, by Jacquemart in 1976, and by Baert et al. in 1993. *T. paschae* has only ever been described on Rapa Nui. The only description of this species was based on a female, and was provided by Berland in [8]. The description focused mostly on highly variable characters (size, color, and eye pattern), but also provided illustrations of the carapace and chelicerae. However, the description does not include any of the key diagnostic features (genitalia). Based on the body measurements that are reported in this description [9], Baert placed it among the largest species of the genus [8]. The third *Tetragnatha* species on Rapa Nui has only recently been observed in a survey of spiders in 2012 by Cotoras et al. [14]. The species is morphologically distinct from *Tetragnatha* species that are known to inhabit other Pacific Islands and Chile (the two most likely sources of human introduction), and it has therefore been hypothesized that it may represent a second endemic *Tetragnatha* species on Rapa Nui [14]. The 2012 survey also found no evidence of *T. paschae* or *T. nitens*, despite covering a wide range of environments and geographic locations. Since the 1993 survey by Baert et al. also found no evidence of *T. paschae* [9], it has been hypothesized that *T. paschae* is now extinct [9,14]. 

Only three specimens that match the description of *T. paschae* were found in our searches of several museum collections (Table S1). The type specimens of the species that were described by Berland appear to have been lost, since they were not found in the Muséum national d'Histoire naturelle in Paris, where Berland was based. All of the specimens originate from Rapa Nui and are in the collection of the Natural History Museum (London, U.K.): three were collected in 1925 by P.H. Johnson, as part of the Pacific expedition of the S.Y. St. George, and one was collected in 1913, probably by Francisco Fuentes from the Museo Nacional de Historia Natural (Santiago, Chile). The three females match the original illustration in terms of the eye distribution and teeth pattern. In vial with P.H. Johnson’s collections, there is also an immature male, but its identification cannot be confirmed because the original description was based only on a female. 

Since *T. paschae* is only known from an incomplete description and museum specimens, our understanding of the identity and evolutionary origins of these Rapa Nui spiders requires the recovery of DNA from preserved specimens. Arachnids remain challenging for ancient DNA analyses, due to their often complicated preservation histories and small sizes. The most common approach to preserve spiders in museums is to store specimens in jars filled with 70% ethanol at room temperature [15]. This liquid environment may vary for a single specimen over its preservation history, as ethanol evaporates and jars are refilled. As the proportion of water in the liquid environment increases, so does the potential for DNA hydrolysis, a common form of DNA decay [16]. Samples may also be stored in or refilled using mixed or alternative liquids, including methanol, isopropyl alcohol, acetone, or methyl ethyl ketone [17]. Different storage and refill histories, which are often impossible to reconstruct, may all influence long-term DNA preservation. Finally, liquid storage in bulk, where different species, genera, and even families are preserved in the same jar, may lead to co-extraction of DNA from different individuals, further complicating genomic reconstruction and analysis. 

Several studies have assessed methods to recover DNA from pin-preserved insects [18,19,20,21], however less work has focused on recovering preserved DNA from specimens that are liquid-preserved [22,23]. In addition to the potential for degradation during liquid storage, museum-preserved insects and spiders are small and often precious, limiting the amount of sample that is available for destructive analysis. Consequently, extraction methods that do not destroy the physical structure of the specimen or that are minimally destructive are often preferred. Recently, an extraction protocol was described that used surfactants and reducing agents to lyse cells, with the goal being to recover DNA from museum-preserved insects without requiring the destruction of the samples [21]. 

Here, we take advantage of recent improvements in the efficiency of DNA recovery from ancient and historic remains [24] to explore the evolutionary histories of *Tetragnatha* spiders from Rapa Nui. We combine the use of the lysis buffer described in [21] on pulverized spider legs with a DNA purification protocol that was developed specifically for ancient DNA that requires only a small amount of input tissue [24]. We use this optimized protocol to generate mitochondrial genomic data from museum-preserved *Tetragnatha*. We compare these data with mitochondrial genomic data from recently collected *Tetragnatha* from Rapa Nui, and field sites in Asia (Myanmar), America (Chile and Peru), and French Polynesia (the Society Islands), to explore the phylogenetic relationships, possible extinction, and recent introduction of *Tetragnatha* spiders on Rapa Nui. 

## 2. Methods

### 2.1. Samples

**Figure 1:**
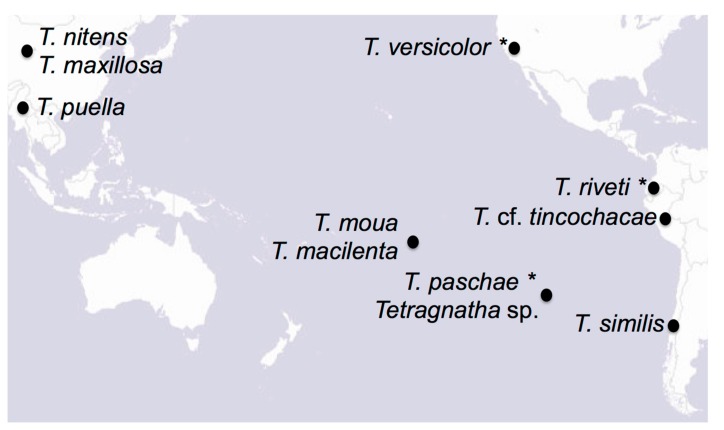
Map of the Pacific Rim with the species included on this study. Names with asterisks correspond to museum specimens. Map from www.esri.com.

We gathered specimens representing nine different *Tetragnatha* species to generate mitochondrial assemblies for phylogenetic analysis (Table 1). We collected six new specimens during recent field expeditions to Rapa Nui, Chile, the Society Islands, Myanmar, and Peru, and selected three specimens from museum collections. We also downloaded two previously published mitochondrial genomes from Asian *Tetragnatha* species from GenBank. We selected these 11 species as they span a wide geographic range within the Pacific Rim (Table 1; Figure 1). We did not have access to the previous *T. nitens* that were collected on Rapa Nui, but we included in the analysis several published sequences for this cosmotropical species. We also downloaded the three most closely related mitochondrial genomes available on GenBank (Araneidae and Lycosidae; Table 1), which we used as outgroups in our phylogenetic analyses. 

### 2.2. DNA Extraction 

We extracted DNA from the six field-collected spiders using the Qiagen DNEasy Tissue Kit, (Venlo, The Netherlands) following the manufacturer protocol. Four legs were removed from the right side of the spider and pulverized in 200 μL of lysis bufffer (180 μL of buffer ATL + 20 μL Proteinase K). We then incubated the samples at 56 °C overnight. After digestion, we purified DNA following the manufacturer’s instructions, and eluted DNA into a final volume of 100 μL.

We extracted DNA from the three museum specimens in a geographically isolated laboratory facility that has been designed for ancient DNA research. We followed standard ancient DNA protocols throughout the experimental process [25], including the use of sterile reagents, consumables, and outerwear.

We followed slightly different extraction protocols for the three museum specimens, based on their rarity and what was known about their preservation history. We performed two different DNA extractions from *Tetragnatha versicolor*. From this specimen, we removed all eight legs. We then extracted DNA from four of these using the Qiagen DNEasy Tissue Kit, following manufacturer’s instructions. For the other four legs, we followed a protocol optimized to recover short fragments of DNA from ancient bone [24]. 

For *T. paschae* and *Tetragnatha riveti* we used a DNA extraction protocol that is based on [21,24]. Our modified protocol used a lysis buffer similar to that described in [21], which was developed for non-destructive DNA extraction from museum insects. Our lysis buffer comprised (in 100 mL) 5.3 mL 1 M Tris-HCl (pH 8.0), 5.3 mL 0.2 M EDTA, 10.6 mL 20% Sarkosyl, 1 mL 2-mercaptoethanol, and distilled water. Sock solutions of this buffer can be wrapped in foil and stored at 4 °C prior to use. At the moment of use, we added 200 μg/mL proteinase K, and incubated tissue overnight at 56 °C. We then purified the lysed DNA via centrifugation, as described in [21], as centrifugal purification has been shown to recover smaller fragments of DNA than purification using silica beads described in [21].

For *T. riveti,* which was stored in a jar with other spider species, we removed four legs from the right side and washed these three times in 500 μL of distilled water for one minute. The aim of this washing step was to remove potential superficial DNA that may originate from the other specimens in the same jar. After washing, we pulverized the four legs in 1 mL of the lysis buffer that is described above. 

For *T. paschae,* because of the rarity of our specimens, we selected to remove and process only two legs from only one of the specimens using a non-destructive method. We selected one of the specimens from 1925, due to its more recent preservation. We placed these two legs in the lysis buffer without pulverization. After overnight digestion, we removed these two legs from the lysis buffer and washed them three times in 70% ethanol, and then transferred them back into their original vial.

We eluted DNA from the three museum spiders into 50 μL of Tris-EDTA (TE) buffer.

### 2.3. Library Preparation and Sequencing

We transformed the DNA extracts into barcoded Illumina sequencing libraries [26]. We then pooled and sequenced all of the libraries on an Illumina MiSeq (San Diego, CA, USA), using 150-cycle v3 chemistry (2 × 75), targeting an initial 300,000 reads per library. After calculating the endogenous DNA content, we sequenced the museum specimens more deeply to compensate for the amount of non-spider DNA. Total depth of sequencing is provided in Table 2. For *T. versicolor*, we prepared one library for each DNA extract. For *T. paschae* and *T. riveti*, we prepared multiple libraries for each species to compensate for poor preservation (specifically, the small number of unique DNA molecules recovered from each library).

### 2.4. Mitochondrial Genome Assembly

We combined data from samples for which more than one library was sequenced, and trimmed adapters and merged reads using a customized version of SeqPrep [27]. We then performed a reference guided mitochondrial assembly using a wrapper script (wormhole Mitochondrial Iterative Assembler, whMIA) to run the program Mitochondrial Iterative Assembler (MIA) [28], with the complete mitochondrial genome of *Tetragnatha maxillosa* (KP306789.1) as the initial reference. For field-collected samples, we mapped reads with the parameters -i -k 13 -w “./mia -c -U –C”–H 8000. For the museum samples, we used similar mapping parameters, but did not use a hard cutoff for the mapping score (-H 8000). The total number of mapped reads and the respective coverage are provided in Table 2.

After mapping, we called consensus mitochondrial genome sequences using two approaches [29]. Our “relaxed” approach required a minimum of 3X coverage, and at least 66% agreement per site in order to call a base. A “strict” approach required a minimum of 10X coverage and 90% agreement per site. Under both of the approaches, sites not meeting these criteria were masked as ‘N’. We assessed the resulting mitochondrial genomes by eye, including mapping genes and checking for stop codons within predicted coding regions. 

### 2.5. Phylogenetic Reconstruction

We created two alignments; one with sequences generated using the “relaxed” approach (Table S2) and the other with sequences generated using the “strict” approach (Table S3). To each alignment, we added the five sequences downloaded from NCBI (Table 1). We aligned each data set using default parameters of MAFFT [30], as implemented in Geneious v. 5.6.7 [31]. We then used the annotated reference genome KP306789.1 to determine the locations of coding sequences, tRNAs and rRNAs. Because it was poorly assembled, we excluded the control region. The “relaxed” data set produced an alignment of 12,737 base-pairs (bp), while the “strict” data set resulted in a 12,291 bp alignment. *T. paschae*, for which we could only generate 242 bp of sequence meeting the “relaxed” base-calling criteria, was only included in the “relaxed” alignment. 

We then reconstructed the phylogenetic relationships between the spider species in both of the alignments using RAxML v. 8.2.4 [32]. For both alignments, we used five data partitions: 1st codon positions; 2nd codon positions; 3rd codon positions; rRNAs; and, tRNAs. Following RAxML’s manual [33], we used the GTR + G model for each partition. *Pirata subpiraticus* (Lycosidae) was used as an outgroup. We estimated support for the inferred topology using 500 bootstrap replicates.

To test the robustness of this topology, we also performed a Bayesian phylogenetic reconstruction using MrBayes v. 3.2.6 [34,35]. Evolutionary models for each partition were chosen using PartitionFinder v. 1.1.1 [36]. For the “relaxed” alignment, the models that were selected were: CDS 1st codon position: GTR + I + G; CDS 2nd position: GTR + I + G; CDS 3rd position: HKY + I + G; rRNA and tRNA: GTR + I + G. For the “strict” alignment the models selected were: CDS 1st codon position: GTR + I + G; CDS 2nd position: GTR + G; CDS 3rd position: HKY + I + G; rRNA and tRNA: GTR + I + G. We performed two runs of four independent chains. Each run consisted of 1,000,000 generations sampling every 1000 generations. At the end of the run, the standard deviation of split frequencies was <0.01. Using Tracer v. 1.7.5, parameter convergence was determined by estimating the Effective Sample Size (ESS) [37]. All parameters had an ESS >200 after removal of burn-in (25%). A consensus tree was constructed after removal of this burn-in.

While the *T. nitens* in our alignment originates from China, the species is known to have a wide circumtropical distribution [38]. We therefore downloaded all available *T. nitens* and *T. moua* mitochondrial sequences from GenBank (EU796911-EU796917, EU796906-EU796908). Our aim was to determine the relationship between the *T. nitens* from China that we used in our main phylogenetic analyses and other *T. nitens* from a variety of geographic locations. Since the available sequences only included the *COI* gene, we estimated a phylogeny of these samples, combined with data from our “strict” alignment, based solely on this gene. Since our *T. paschae* sequence did not include any sites from the *COI* gene, it had to be excluded from this analysis. We also excluded any sequences with large numbers of missing sites for this gene, and then estimated a phylogeny using RAxML, as above.

All of the trees were visualized using FigTree v. 1.4.0 [39].

## 3. Results

### 3.1. DNA Recovery

We recovered DNA from all nine specimens for which we attempted DNA extraction. The amount of recovered DNA varied between samples, and, as expected, less DNA was recovered from the museum-preserved specimens than from the present-day specimens. 

### 3.2. Mitochondrial Genome Assemblies

We assembled partial mitochondrial genomes from all nine spiders, although we were only able to recover a small (242 bp) fragment from the oldest and most poorly preserved specimen, *T. paschae*. We deposited the resulting “relaxed” partial mitochondrial genomes and the genes *COI* and *ND4* from *T. paschae* in GenBank, with accession numbers (MG564492–MG564500, MG597260). The genes *COI* and *ND5* of *T. paschae* and all of the “strict” assemblies are in Supplementary file 2. Details of the sequencing results are provided in Table 2. 

### 3.3. Phylogenetic Reconstruction

**Figure 2:**
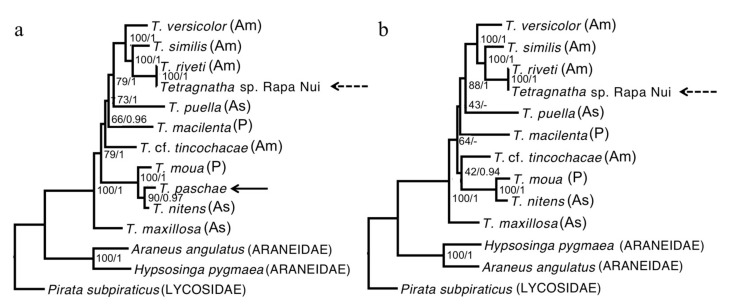
Maximum likelihood reconstruction. (**a**) Relaxed consensus including *T. paschae*. (**b**) Strict consensus not including *T. paschae.* The node values represent the bootstrap support/Bayesian posterior probability. As: Asia; Am: America; P: Pacific.

**Figure 3:**
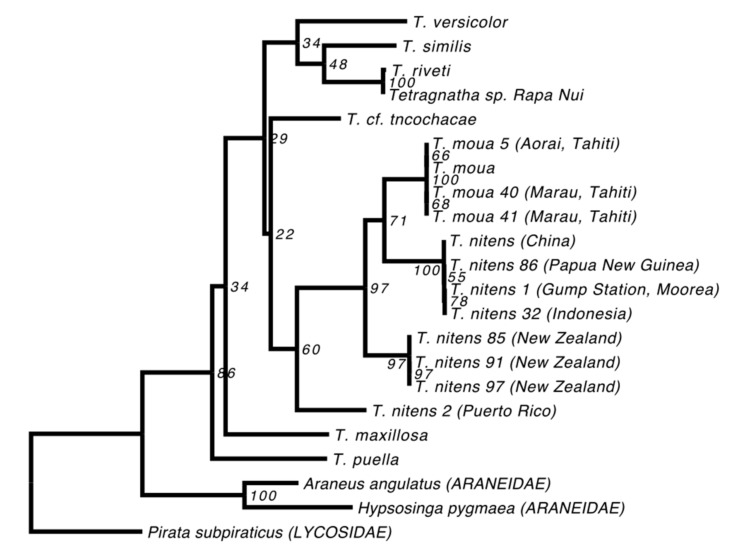
Maximum likelihood reconstruction including extra populations of *T. nitens* and specimens of *T. moua*. The reconstruction was done using the strict consensus sequences of *COI*. The *T. moua* and *T. nitens* specimens marked with asterisk correspond to the ones used on Figure 2.

The phylogenies we estimated using our “relaxed” and “strict” alignments were consistent; although not all of the nodes could be resolved, all that received high support were observed in both reconstructions (Figure 2). We also found that the topologies were robust to our use of different methods of reconstruction and evolutionary models (Figure 2). All of the *Tetragnatha* species that were included in our analyses form a strongly supported monophyletic group. Although *T. paschae* (indicated by an arrow on Figure 2) is represented by only a small fragment of DNA, we were able to establish that it falls within a strongly supported clade with the Tahitian endemic species *T. moua,* and sister to a *T. nitens* specimen from China. In the *COI* gene phylogeny (Figure 3), which includes more specimens from *T. nitens*, we found that the *T. nitens* and *T. moua* specimens that are described in Figure 2 fall within a larger clade of *T. nitens*, which includes other specimens from Asia (Papua New Guinea and Indonesia) and the Pacific (Mo’orea, Society Islands). The *Tetragnatha* sp. from Rapa Nui (indicated by an arrow with dashed line on Figure 2) appears as a sister to *T. riveti,* and is nested on a clade with other American species. 

## 4. Discussion

Our phylogenies establish that the modern unidentified *Tetragnatha* from Rapa Nui has a distinct evolutionary origin from *T. paschae*. The modern *Tetragnatha* sp. is most closely related to *T. riveti* from Ecuador, and falls within a strongly supported clade with species from Chile and the United States, while, *T. paschae* is nested within the *T. nitens* species complex.

### 4.1. Revealing the History of Tetragnatha Species on Rapa Nui

Because of its geographic isolation and relatively recent history of colonization by humans, the biotic communities on Rapa Nui comprise mixtures of colonizing species originating from disparate geographic locations at different times [9,14,40,41]. The island therefore provides an opportunity to study dispersal and introduction patterns. This study used *Tetragnatha* spiders, a previously unexplored component of Rapa Nui’s biotic community, to study these patterns. We used ancient DNA approaches and mitochondrial phylogenetic reconstructions to explore the relationships between *T. paschae* and the *Tetragnatha* species that were collected on Rapa Nui in 2012, and other *Tetragnatha* from the surrounding geographic region.

Due to the poor preservation of the *T. paschae* specimens, we were able to reconstruct only a small fragment of the mitochondrial genome. Nevertheless, our results confirm that *T. paschae* and the newly discovered *Tetragnatha* belong to distinct evolutionary lineages, with independent colonization histories (Figure 2). 

The genetic similarity between *T. riveti* and *Tetragnatha* sp. Rapa Nui suggests that the latter was only recently introduced to Rapa Nui (Figure 2). This is supported by the failure of previous surveys of Rapa Nui to observe this species [9]. Moreover, it is unlikely to have been misidentified in Berland’s description of the Swedish Expedition (1916–1917) [8], since Berland himself described *T. riveti* prior to publishing his work on Rapa Nui [42]. Our results led us to undertake a morphological comparison of these species, which revealed similarities in taxonomically relevant characters. In particular, both of the specimens share three very distinctive characteristics of the male chelicerae: (1) a median strong tooth in the promargin; (2) next to this strong tooth, a small promarginal tooth directed towards the proximal end of the chelicerae; and, (3) a dorsal spur (thorn-like projection) on the distal end. The male palp also has several distinctive traits that are common to both specimens, including a straight embolus and oval-shaped bulbus. The anterior and posterior eyes in both samples are arranged in two curved and opposite rows. Finally, they both have elongated and similarly shaped endites. These morphological similarities were not discovered in the previous analysis of the Rapa Nui specimen [14] because comparisons were only made with species that were described in Chile and Polynesia; and *T. riveti* has only been described for Ecuador [42]. 

Given the morphological similarities and the short length of the branch separating these two species in the mitochondrial phylogeny, it seems likely that the recently collected *Tetragnatha* from Rapa Nui is *T. riveti,* rather than a new endemic species. While *T. riveti* has only been reported for Ecuador [42], the full range of the species has not been investigated and there is no comprehensive revision for the genus in the region. In particular, it is not known whether the species’ range extends to Peru or Chile, countries that historically have had more contact with Rapa Nui than Ecuador and are therefore more likely routes of anthropogenic introduction [7]. Based on reported species distributions, fewer than 10% of known arachnid introductions to Rapa Nui are of South American origin, with the majority of the introduced species representing groups with circumtropical or circumglobal distributions [14]. 

**Figure 4:**
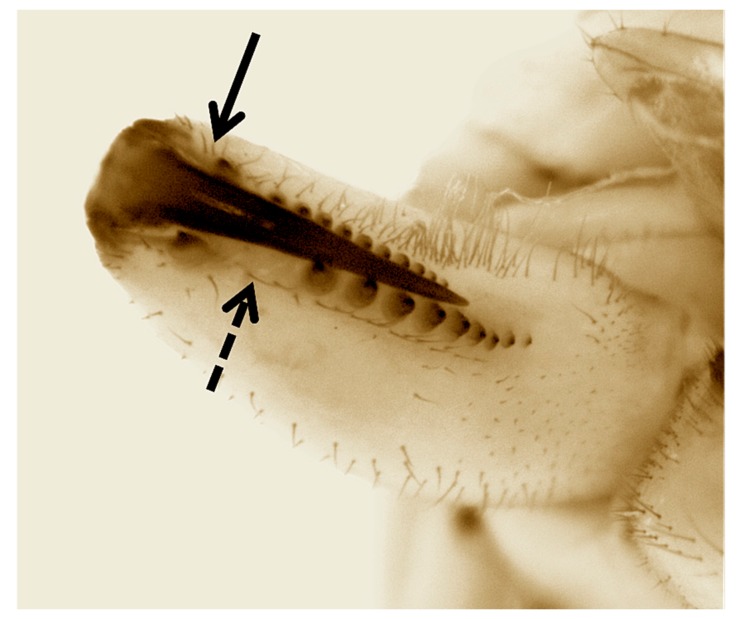
Inner view of left chelicerae of the sequenced female *T. paschae.* Complete arrow indicates the lack of the posterior cusp on the fang. Dashed arrow shows the absence of an enlarged and isolated tooth in the distal portion of the promarginal row of teeth. Both characters are present in *T. nitens.*

Our phylogenies place *T. paschae* within the genetic diversity of *T. nitens*, a species that comprises a complex of highly structured populations [43]. While this might mean that *T. paschae* was incorrectly described as a distinct species, *T. moua*, a Tahitian endemic, also falls within the genetic diversity of *T. nitens* [11]. *T. nitens* presents strong morphological conservation across divergent genetic lineages, and *T. moua*, while falling within the genetic diversity of *T. nitens*, has a distinct genital morphology and cheliceral armature [44]. This divergence from *T. nitens* could reflect local adaptation or an increased influence of random drift due to a small population size. We also observed some phenotypic divergence from *T. nitens* in *T. paschae*. The chelicerae of the sequenced *T. paschae* is missing the posterior cusp on the fang, which is a diagnostic character of females of *T. nitens* [8,45]. It also differs in not having an enlarged and isolated tooth in the distal portion of the promarginal row of teeth [8,45] (Figure 4). However, since we have no description of the male *T. paschae*, and no adult male specimens, we cannot fully assess the morphological divergence between *T. paschae* and *T. nitens*. Also, because of the small amount of mitochondrial sequence that we were able to generate for *T. paschae,* and the lack of specimens of *T. nitens* from Rapa Nui, we cannot fully evaluate the evolutionary distance of *T. paschae* from *T. nitens* and *T. moua*. 

If *T. paschae* was a distinct species that is now globally extinct, it would be the first extinct arachnid from which DNA has been amplified, and a rare description of a spider extinction. The IUCN red list currently includes only three extinct spider species, all from the Seychelles Islands [46,47]. This number of arachnid species that are recently extinct is likely to be much greater than this [48], and the paucity of described spider extinctions highlights the importance of surveys and monitoring of species, particularly island endemics. The causes and temporal dynamics of arthropod extinctions, in general, are poorly understood, and the further application of ancient DNA techniques to museum collections of arthropods and comparisons with extant species is a promising direction for future research [49].

### 4.2. Recovering Ancient DNA from Museum-Preserved Spiders

Our results show the combined power of field surveys and molecular analysis of museum-preserved samples to identify cryptic species and infer evolutionary relationships. However, our results also highlight the challenges of performing ancient DNA research on museum-preserved spiders. For example, the amount of recovered endogenous DNA varied considerably between the museum-preserved samples that are processed here (Table 2). Moreover, interpreting this is complicated by both the paucity of comparative molecular data from living species [50] and incomplete records of the preservation history of the specimens.

Our approach, which combines two previously published optimizations for ancient DNA [21,24] was successful in recovering DNA from very small amounts of a nearly 100 year-old spider. A previous comparison of DNA extraction methods for bone powder showed that only proteinase K and EDTA in the lysis buffer positively influenced DNA recovery [51]. For bone powder, the utility of EDTA is known to be its decalcifying effect, as preserved DNA in bone tends to form a complex with hydroxyapatite [52]. However, this does not apply to spiders, where the majority of recoverable DNA is probably preserved within cells. Based on a recently published non-destructive method for DNA recovery from museum-preserved insects [21], we included in our lysis buffer the surfactant Sarkosyl, to dissolve cellular membranes, and the reducing agent 2-mercaptoethanol, to break S-S bonds. We selected 2-mercaptoethanol instead of dithiothreitol (DTT), due to its longer half-life (>100 h 2-mercaptoethanol vs. 4 h DTT) in conditions similar to those of the lysis buffer (pH 8.5, 20 °C and 1.0 mM EDTA) [53]. We note, however, that an advantage of DTT is that the reduction of the S-S bonds is through an irreversible reaction, instead of an equilibrium reaction where the reducing agent needs to be in excess in order to move the reaction towards the reduced products [54]. To increase yield further, we combined the approach from [21] with modified DNA purification columns, as described in [24]. Although the size of individual specimens limits the feasibility of direct comparative analyses, our results suggest that the combination of these two approaches is an appropriate choice for the recovery of DNA from museum-preserved spiders. 

Another challenge to ancient DNA research in spiders is the paucity of comparative genomic data from living species. Currently, only four spider genomes are published (*Stegodyphus mimosarum*, *Acanthoscurria geniculata*, *Nephila clavipes,* and *Parasteatoda tepidariorum* [55,56,57]). Since spiders originated at least 340 Million years ago (Ma) [58], these four species are not likely to be ideal references for read-mapping or calculating endogenous content for the majority of preserved specimens. However, promising developments in both genome sequencing and assembly technologies [59,60,61] are likely to increase the pace and diversity of published spider genomes, despite the sequencing challenges inherent to this taxonomic group [50]. These resources will accelerate spider research and enable more efficient and powerful research using spiders that are preserved in museum collections, as well as other environments, including lake sediments [62], sinkholes [63], permafrost [20,64], and archeological sites [65]. 

## 5. Conclusions

We assembled partial mitochondrial genomes from museum-preserved and field-collected spiders, using a modified DNA extraction approach. Using these data, we showed that the only endemic spider that has been described on Rapa Nui, *T. paschae* is nested within the *T. nitens* species complex. This brings into question whether *T. paschae* is truly distinct from *T. nitens*, and therefore whether its absence from recent surveys of Rapa Nui might reflect an extinction. We found that *T. paschae* is evolutionarily distant from the *Tetragnatha* species found in the most recent survey of Rapa Nui. Our results suggest that this recently observed species is most likely *T. riveti*, which is a species that has previously only been found in Ecuador, and that this species was brought to the island only recently as a human introduction. Our results show the combined power of field surveys and ancient DNA analysis of museum specimens to reveal phylogenetic and dispersal history of rare and extinct spiders.

## Figures and Tables

**Table 1:** Species included on the study.

Species Name	Geographic Origin	Collection/GenBank	Preservation Conditions	Collection Year
*Tetragnatha paschae*	Rapa Nui	Natural History Museum of London	Ethanol 70% originally and currently 80% IDA (industrial denatured alcohol), room temperature	1925
*Tetragnatha riveti*	Ecuador	California Academy of Sciences	Ethanol 70%, room temperature	1943
Tetragnatha versicolor *	United States (California)	California Academy of Sciences	Ethanol 70%, room temperature	1947
*Tetragnatha puella*	Myanmar	California Academy of Sciences	Ethanol 95%, −20 °C	2003
*Tetragnatha* cf. *tincochacae*	Perú	California Academy of Sciences	Ethanol 95%, −20 °C	2010
*Tetragnatha* sp.	Rapa Nui	This study	Ethanol 95%, −20 °C	2012
*Tetragnatha similis*	Chile	This study	Ethanol 95%, −20 °C	2015
*Tetragnatha macilenta*	Tahiti	This study	Ethanol 95%, −20 °C	2015
*Tetragnatha moua*	Tahiti	This study	Ethanol 95%, −20 °C	2015
*Tetragnatha maxillosa*	China	KP306789.1	-	-
*Tetragnatha nitens*	China	KP306790.1	-	-
*Hypsosinga pygmaea*	China	NC028078.1	-	-
*Araneus angulatus*	China	KU365988.1	-	-
*Pirata subpiraticus*	China	KM486623.1	-	-

* Morphologically identified as *Tetragnatha laboriosa*, but cytochrome oxidase I (*COI*) data shows that it is more closely related to *Tetragnatha versicolor*. For more information about these specimens, see the metadata associated with the GenBank sequences.

**Table 2:** Species included on the study.

*Species Name*	Merged Reads	Unmerged Read Pairs	Mapped Reads	% Mapped Reads	Coverage *
*T. paschae*	2,035,294	127,651	274	0.012	1.142
*T. riveti*	1,152,000	191,722	1717	0.112	7.754
*T. versicolor*	1,664,113	81,418	19,793	1.08	96.616
*T. puella*	203,936	464,142	4695	0.415	27.776
*T.* cf. *tincochacae*	205,455	501,781	2811	0.232	16.923
*Tetragnatha* sp. Rapa Nui	187,032	411,508	2944	0.291	17.622
*T. similis*	109,240	580,116	2003	0.158	11.481
*T. macilenta*	159,505	729,383	2023	0.125	11.866
*T. moua*	307,068	732,206	3663	0.207	21.920

* Coverage before application of “Strict”/“Relaxed” filter and removal of misassembles.

## Supplementary Materials

The following are available online at www.mdpi.com/2073-4425/8/12/403/s1, Table S1: List of museums visited; Table S2: Genes used in the Relaxed phylogeny; Table S3: Genes used in the Strict phylogeny; Supplementary file 2: Strict consensus sequences + *ND5* and *COIII* from *T. paschae*.
