# Supplementary material for: Ancient DNA Resolves the History of Tetragnatha (Araneae, Tetragnathidae) Spiders on Rapa Nui
Source: Genes (Basel). 2017 Dec 21;8(12):403. doi: 10.3390/genes8120403 (PMC5748721; doi:10.3390/genes8120403)
Supplement: Supplementary file 1 [file genes-08-00403-s001.zip › Supplementary files/Supplementary_file_1.docx]

Supplementary information for:

**Ancient DNA resolves the history of** ***Tetragnatha* spiders on Rapa Nui**

Darko D. Cotoras, Gemma G. R. Murray, Joshua Kapp, Rosemary Gillespie, Charles Griswold, W. Brian Simison, Richard E. Green and Beth Shapiro

Table S1: List of museums visited

| **Country** | **City** | **Museum** |
| --- | --- | --- |
| Chile | Santiago | Museo Nacional de Historia Natural |
| Costa Rica | Heredia | Instituto Nacional de Biodiversidad |
|  | San José | Universidad de Costa Rica |
| Denmark | Copenhagen | Zoologisk Museum |
| France | Paris | Muséum national d'Histoire naturelle |
| Germany | Frankfurt | Senckenberg Museum |
| Panamá | Ciudad de Panamá | Museo de Invertebrados G.B. Fairchild |
| United Kingdom | London | Natural History Museum |
| United States | San Francisco, CA | California Academy of Sciences |
|  | Berkeley, CA | Essig Museum of Entomology |
|  | Cambridge, MA | Museum of Comparative Zoology |
|  | Honolulu, HI | The Bernice Pauahi Bishop Museum |

*We also requested specimens to Naturalis (Leiden, The Netherlands)

Table S2: Genes used in the Relaxed phylogeny

|  | *T. paschae* | *T. riveti* | *T. versicolor* | *T. puella* | *T.* cf. *tincochacae* | *Tetragnatha* sp. Rapa Nui | *T. similis* | *T. moua* | *T. macilenta* |
| --- | --- | --- | --- | --- | --- | --- | --- | --- | --- |
| *ND2* |  | x | x | x | x | x | x |  | x |
| *COI* |  | x | x | x | x | x | x | x | x |
| *COII* | x | x | x | x | x | x | x | x | x |
| *ATP6* |  | x | x | x | x | x | x | x | x |
| *COIII* | x | x | x | x | x | x | x | x | x |
| *ND5* | x | x | x | x | x | x | x | x | x |
| *ND4* | x | x | x | x | x | x | x | x | x |
| *ND4L* |  | x | x | x | x | x | x | x | x |
| *ND6* |  | x | x |  | x | x | x | x | x |
| *CYTB* |  | x | x | x | x | x | x | x | x |
| *tRNA-Met* |  |  | x | x | x | x |  |  | x |
| *tRNA-Tyr* |  | x | x | x | x | x | x | x | x |
| *tRNA-Cys* |  | x | x | x | x | x | x | x | x |
| *tRNA-Lys* |  | x | x | x | x | x | x | x | x |
| *tRNA-Asp* |  | x | x | x | x | x | x | x | x |
| *tRNA-Leu* |  | x | x | x | x | x | x | x | x |
| *tRNA-Asn* |  | x | x | x | x | x | x | x | x |
| *tRNA-Ala* |  | x | x | x | x | x | x | x | x |
| *tRNA-Ser* |  | x | x | x | x | x | x | x | x |
| *tRNA-Arg* |  | x | x | x | x | x | x | x | x |
| *tRNA-Glu* |  | x | x | x | x | x | x | x | x |
| *tRNA-Phe* |  | x | x | x | x | x | x | x | x |
| *tRNA-His* |  |  | x | x | x | x | x | x | x |
| *tRNA-Pro* |  | x | x | x | x | x | x | x | x |
| *tRNA-Ile* |  | x | x | x | x | x | x | x | x |
| *tRNA-Ser* |  | x | x | x | x | x | x | x | x |
| *tRNA-Thr* |  | x | x | x | x | x | x | x | x |
| *tRNA-Leu* |  | x | x |  | x |  |  | x | x |
| *tRNA-Val* |  | x | x |  | x |  |  | x | x |
| *tRNA-Gln* |  |  | x |  | x |  |  | x | x |
| *16S* |  | x | x |  | x |  |  | x | x |
| *12S* |  |  | x |  | x |  |  | x | x |

Table S3: Genes used in the Strict phylogeny

|  | *T. riveti* | *T. versicolor* | *T. puella* | *T.* cf. *tincochacae* | *Tetragnatha* sp. Rapa Nui | *T. similis* | *T. moua* | *T. macilenta* |
| --- | --- | --- | --- | --- | --- | --- | --- | --- |
| *ND2* | x | x | x | x | x |  |  | x |
| *COI* | x | x | x | x | x | x | x |  |
| *COII* | x | x | x | x | x | x | x | x |
| *ATP6* | x | x | x | x | x | x | x | x |
| *COIII* | x | x | x | x | x | x | x | x |
| *ND5* | x | x | x | x | x | x | x | x |
| *ND4* | x | x | x | x | x | x |  | x |
| *ND4L* | x | x | x | x | x | x | x | x |
| *ND6* | x | x |  | x | x | x | x | x |
| *CYTB* | x | x | x | x | x | x | x | x |
| *tRNA-Lys* |  | x | x | x | x | x | x | x |
| *tRNA-Asp* | x | x | x | x | x | x | x | x |
| *tRNA-Leu* | x | x | x | x | x |  | x | x |
| *tRNA-Asn* | x | x | x | x | x |  | x | x |
| *tRNA-Ala* | x | x | x | x | x | x | x | x |
| *tRNA-Ser* | x | x | x | x | x | x | x | x |
| *tRNA-Arg* | x | x | x | x | x | x | x | x |
| *tRNA-Glu* | x | x | x | x | x |  | x | x |
| *tRNA-Phe* | x | x | x | x | x |  | x | x |
| *tRNA-His* |  | x | x | x | x | x | x | x |
| *tRNA-Pro* | x | x | x | x | x | x | x | x |
| *tRNA-Ile* | x | x | x | x | x | x | x | x |
| *tRNA-Ser* | x | x | x | x | x | x | x | x |
| *tRNA-Thr* | x | x | x | x | x | x | x | x |
| *tRNA-Leu* |  | x |  | x |  |  | x | x |
| *tRNA-Val* | x | x |  | x |  |  | x | x |
| *tRNA-Gln* |  | x |  | x |  |  | x | x |
| *16S* | x | x |  | x |  |  | x | x |
| *12S* |  | x |  | x |  |  | x | x |
